# Identification and Characterization of *Fusarium* Species Causing Watermelon Fruit Rot in Northern Thailand

**DOI:** 10.3390/plants12040956

**Published:** 2023-02-20

**Authors:** Wipornpan Nuangmek, Jaturong Kumla, Surapong Khuna, Saisamorn Lumyong, Nakarin Suwannarach

**Affiliations:** 1 Faculty of Agriculture and Natural Resources, University of Phayao, Phayao 56000, Thailand; 2 Research Center of Microbial Diversity and Sustainable Utilization, Chiang Mai University, Chiang Mai 50200, Thailand; 3 Department of Biology, Faculty of Science, Chiang Mai University, Chiang Mai 50200, Thailand; 4 Academy of Science, The Royal Society of Thailand, Bangkok 10300, Thailand

**Keywords:** fruit rot, fungal disease, *Fusarium*, pathogen identification, watermelon disease

## Abstract

Fruit rot caused by phytopathogenic fungi is one of the major diseases affecting watermelons (*Citrullus lanatus*) around the world, which can result in unmarketable fruits and significant economic losses. Fruit rot was observed on watermelons throughout the postharvest storage periods in Phayao Province, northern Thailand in 2022. For the present study, a total of ten fungal isolates were isolated from the rot lesions of watermelons. All obtained fungal isolates were then characterized in terms of their pathogenicity. The results indicated that only four fungal isolates caused rot disease with similar symptoms during the postharvest storage period. Based on their morphological characteristics, these four fungal isolates were identified as belonging to the genus *Fusarium*. Using multi-gene phylogenetic analyses with a combination of the translation elongation factor 1-alpha (*tef-1*), calmodulin (*cam*), and RNA polymerase second largest subunit (*rpb2*) genes, the fungal isolates were subsequently identified as *Fusarium compactum* and *F. paranaense*. Taken together, the results of this study indicate that *F. compactum* and *F. paranaense* cause fruit rot disease in watermelons. To the best of our knowledge, this is the first study to report *F. compactum* and *F. paranaense* as novel pathogens of watermelon fruit rot both in Thailand and elsewhere in the world.

## 1. Introduction

Watermelon (*Citrullus lanatus*) is one of the most significant economic fruits within the family Cucurbitaceae. It has successfully been planted and farmed in subtropical and tropical regions throughout the world [1,2,3]. In 2022, the Food and Agriculture Organization Statistical Database (FAOSTAT) [4] demonstrated that China was the world’s largest producer of watermelons producing 60.25 million tonnes (with global production recorded at 101.62 million tonnes), followed by Turkey, India, Iran, and Algeria. This increasing trend in watermelon production is expected to continue into the future. In Southeast Asia, watermelon production in the area is led by Vietnam followed by Indonesia, the Lao People’s Democratic Republic, Thailand, and the Philippines [4]. Many scientific studies have reported that watermelon fruits are a good source of nutrition for humans. They contain a variety of important nutrients, including amino acids, carbohydrates, fiber, minerals, organic acids, proteins, sugars, and vitamins [5,6,7]. Furthermore, watermelon fruits contain several beneficial chemical compounds, including alkaloids, flavonoids, glycosides, phenols, tannins, terpenoids, saponins, and steroids, which possess advantageous pharmacological properties [8,9]. These compounds can be utilized in therapeutic approaches due to their antimicrobial, anticancer, antiulcer, antioxidant, anti-inflammatory, antihypertensive, analgesic, and antigiardial properties, which allow them to function against prosthetic hyperplasia and serve as atherosclerosis, gastroprotective, hepatoprotective, and laxative agents [8,9,10,11].

In Thailand, watermelon is currently an economical crop, and the area of cultivation for watermelons is continually increasing [12]. The majority of watermelon production in the northern region is located in the Provinces of Chiang Mai, Phayao, Kamphaeng Phet, Phichit, Sukhothai, and Phitsanulok [13]. Watermelon is cultivated and harvested twice a year in Thailand (from January to May and from mid-October to December). The damage to watermelons caused by fruit rot diseases can result in significant losses for farms in terms of productivity and quality [14]. Watermelons can be affected by a variety of diseases caused by bacteria, fungi, and viruses throughout the growing season, harvest procedure, and postharvest storage period [13,15,16]. Fruit rot disease is known to be the most common and widespread disease in watermelon fruits during the pre and postharvest periods (e.g., storage and transportation) [17,18,19]. This disease can be caused by a number of fungal pathogens within the genera *Aspergillus*, *Alternaria*, *Fusarium*, *Curvularia*, *Macrophomina*, *Phytophthora*, *Lasiodiplodia*, *Sclerotium*, and *Pythium* [13,20,21,22,23,24,25]. The symptoms are characterized by the presence of spots, water-soaked lesions, and often depressions. The lesions enlarge gradually, eventually covering most of the entire fruit. Then, the insides of the infected fruit are completely decayed [19,26]. Due to the formation of water-soaked lesions on the fruit surface, rot disease reduces the quality of the fruit and causes them to appear unattractive to consumers, which significantly reduces their market value [27]. 

Several synthetic fungicides are commonly used to prevent disease infections in watermelons in order to safeguard crop yield and quality, as they are typically affordable, easy to apply, and effective [28,29]; for example, copper hydroxide, cyazofamid, dimethomorph, ethaboxam, fluopicolide, mandipropamid, mefenoaxam, oxathiapiprolin, phthalimide, and potassium phosphite have been used to control fungi causing fruit rot disease in watermelons by spraying [19,27,30]. However, it has been widely recognized that such synthetic fungicides are hazardous to the environment, the health of farmers and consumers, and may contribute to the development of fungicide-resistant strains [31,32].

The global demand for watermelon fruit continues to increase in accordance with the rapid growth of the world’s population [6] resulting in a significant increase in the cultivation area of watermelons. However, the practice of growing crops in unsuitable environments has also increased the prevalence and severity of certain fungal diseases [33,34]. In the context of this study, fruit rot disease was observed in watermelons during the two postharvest storage period phases in Phayao province, northern Thailand in the year 2022 (February to May and mid-October to December) with a percentage of affected fruits that ranges between 15% and 20%. Consequently, a significant amount of that fruit crop became unmarketable. Consequently, the aim of this investigation was to isolate the causative fungi responsible for this disease. The obtained fungi were characterized and identified using a combination of morphological features and molecular data. Pathogenicity tests were performed, and Koch’s postulates were used to evaluate the asymptomatic watermelon fruits with isolated fungi.

## 2. Results

### 2.1. Disease Symptoms

**Figure 1:**
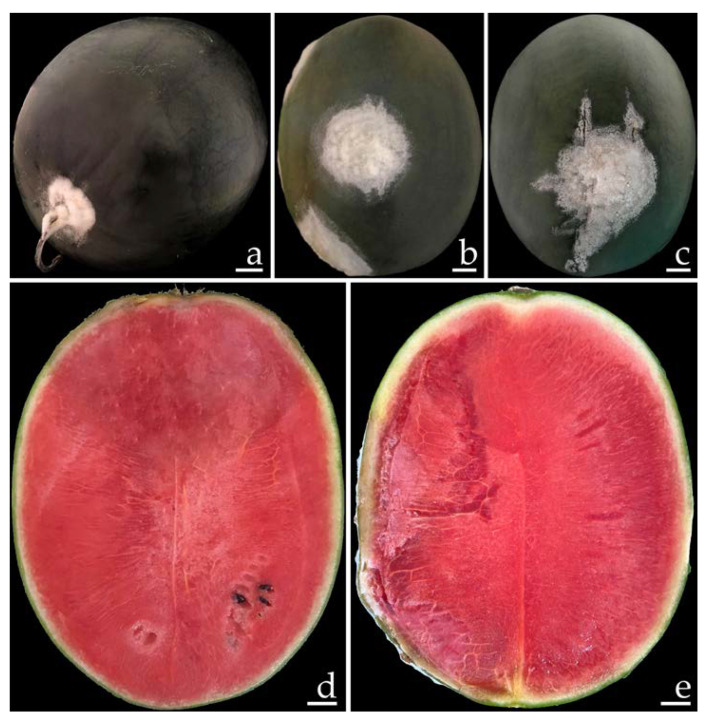
Naturally occurring symptoms of fruit rot in watermelon (**a**–**c**). A cross section of the infected watermelon fruits shows the internal decaying areas (**d**,**e**). Scale bars: (**a**) = 30 mm; (**b**,**c**) = 20 mm; (**d**,**e**) = 15 mm.

Ten samples of watermelon with fruit rot were collected from the markets in Phayao Province, northern Thailand for each time period. The primary symptoms of disease during the postharvest storage period appeared as green bruised spots on the watermelon fruits (Figure 1a). These spots then grew into dark green bruised spots surrounded by white mycelia (Figure 1b,c). One week after collection, the infected fruits exhibited mild to moderate (22–40% of disease infection on fruit areas) infection by rot symptoms. The lesions on the watermelon fruit gradually expand and combine to encompass the whole fruit, providing the infected fruit with a bruised, decayed, and broken appearance. The internal area of decomposition seemed obviously degraded and the surrounding tissues were soaked with water (Figure 1d,e).

### 2.2. Fungal Isolation

Ten fungal isolates (FPY1 to FPY10) were isolated from the watermelons that were collected and which exhibited the typical rot symptoms. Subsequently, all fungal isolates were inoculated into asymptomatic commercial watermelons. The conidia collected from two-week-old cultures on potato dextrose agar (PDA) of each fungal isolate were used as the inoculum. A conidial suspension of each fungal isolate was individually dropped onto the wounded fruits at the equator of each fruit. After three days of conidial inoculation, only four fungal isolates—namely, FPY1, FPY4, FPY7, and FPY9—led to the development of rot lesions. The disease symptoms of these four fungal isolates are provided below. All four fungal causal agents—namely, FPY1, FPY7, FPY4, and FPY9—were stored in 20% glycerol and submitted to the culture collection of the Sustainable Development of Biological Resources (SDBR-CMU), Faculty of Science, Chiang Mai University, Thailand, with the numbers SDBR-CMU461, SDBR-CMU462, SDBR-CMU463, and SDBR-CMU464, respectively. These four fungal isolates were selected for further species identification.

### 2.3. Morphological Observations

Four fungal isolates (SDBR-CMU461, SDBR-CMU462, SDBR-CMU463, and SDBR-CMU464) were selected and used in this experiment. Fungal colonies of each isolate were observed on oatmeal agar (OA), PDA, and synthetic nutrient-poor agar (SNA) at 25 °C for one week. According to the fungal colony characteristics, the isolate SDBR-CMU461 was related to the isolate SDBR-CMU462, whereas the isolate SDBR-CMU463 was related to the isolate SDBR-CMU464. All fungal isolates produced both macro- and micro-conidia, as well as chlamydospores. Based on these morphological features, all the isolated fungi were initially determined to be members of the genus *Fusarium* [35,36,37,38]. The identification of the fungi was subsequently confirmed by multi-gene phylogenetic analyses. 

### 2.4. Phylogenetic Analysis

The sequences derived from the four fungal isolates obtained in this investigation were submitted to the GenBank database (Table 1 and Table 2). Based on the BLAST results, two fungal isolates—namely, SDBR-CMU461 and SDBR-CMU462—belonged to the *F. incarnatum-equiseti* species complex, whereas the fungal isolates SDBR-CMU463 and SDBR-CMU464 belonged to the *F. solani* species complex. Fungal identification was further confirmed through subsequent multi-gene phylogenetic analyses. Two phylogenetic trees (for *F. incarnatum-equiseti* and *F. solani* species complexes) were constructed in this study. The results of both phylogenetic analyses revealed that the topological results of both the maximum likelihood (ML) and Bayesian inference (BI) analyses employed in each analysis were similar (data not shown). Consequently, the phylogenetic trees generated by the ML analysis are presented.

**Figure 2:**
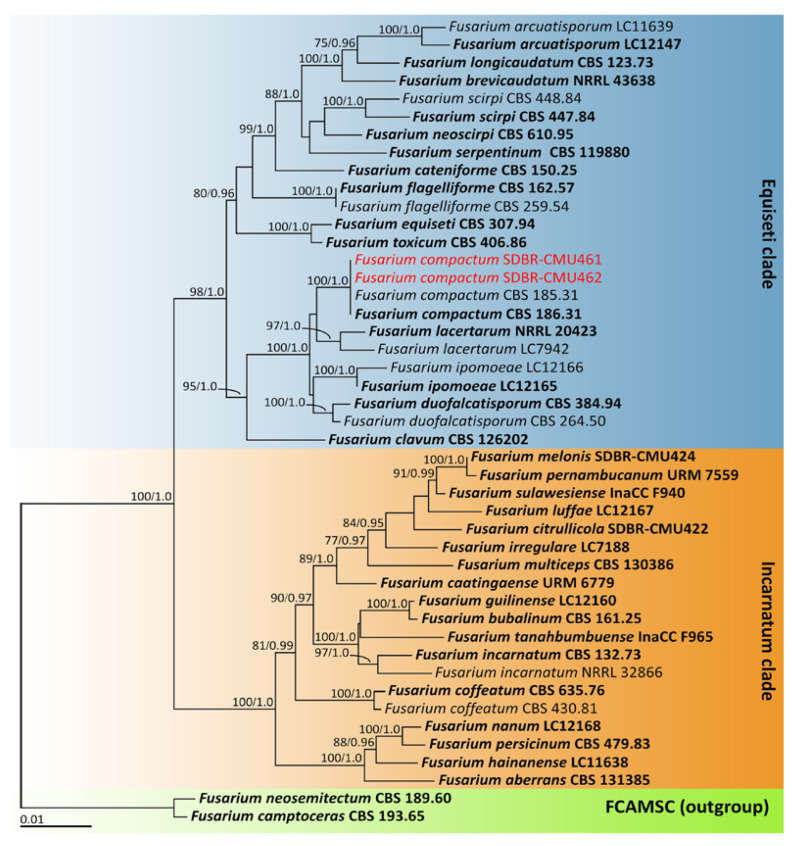
Phylogenetic tree derived from maximum likelihood analysis of *Fusarium incarnatum-equiseti* species complex of the combined *tef-1*, *cam* and *rpb2* sequences of 45 taxa. The outgroup included *F. camptoceras* CBS 193.65 and *F. neosemitectum* CBS 189.60. Numbers above branches are the bootstrap percentages (left) and Bayesian posterior probabilities (right). Branches with bootstrap and Bayesian posterior probabilities values greater than or equal to 75% and 0.95, respectively, are shown at each branch. The scale bar displays the expected number of nucleotide substitutions per site. The sequences of the fungal species derived in this study are shown in red. Type species are shown in bold.

For phylogenetic analysis of the *F. incarnatum-equiseti* species complex, the combined *tef-1*, *cam*, and *rpb2* sequence data set was used, according to the identification techniques used in previous studies [35,36,37,38]. The aligned data set contained 2181 bp including gaps (*tef-1*: 1–704, *cam*: 705–1288, and 127 *rpb2*: 1289–2181) with 45 taxa. The outgroup consisted of *F. camptoceras* and *F. neosemitectum* from the *F. camptoceras* species complex (FCAMSC). A phylogenetic tree is represented in Figure 2. Our phylogenetic tree was constructed with the aim of having similar outcomes to previous phylogenetic studies [13,35,36,37,38]. The phylogenetic tree assigned the two fungal isolates (SDBR-CMU461 and SDBR-CMU462) assessed in this investigation within the same clade of *F. compactum*, which consisted of the type species CBS 186.31 in the *F. equiseti* clade. This clade established a monophyletic clade with high statistical support (100% BS and 1.0 PP). *Fusarium compactum* formed a species that was phylogenetically related to *F. lacertarum*. Therefore, these two fungal isolates (SDBR-CMU461 and SDBR-CMU462) were identified as *F. compactum*.

**Figure 3:**
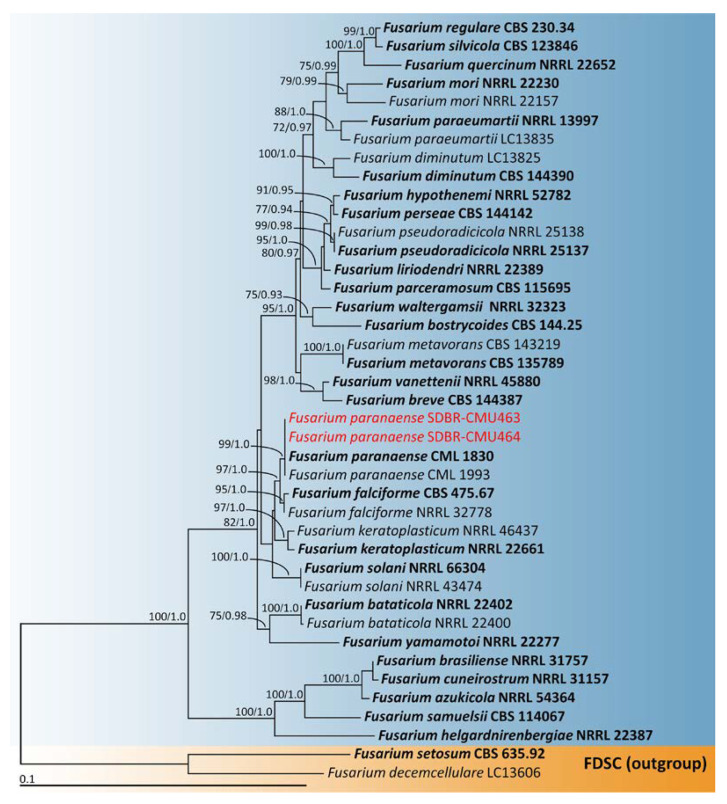
Phylogenetic tree derived from maximum likelihood analysis of *Fusarium solani* species complex of the combined *tef-1* and *rpb2* sequences of 41 taxa. The outgroup included *F. decemcellulare* LC13606 and *F. setosum* CBS 635.92. Numbers above branches are the bootstrap percentages (left) and Bayesian posterior probabilities (right). Branches with bootstrap and Bayesian posterior probabilities values greater than or equal to 75% and 0.95, respectively, are shown at each branch. The scale bar displays the expected number of nucleotide substitutions per site. The sequences of the fungal species derived in this study are shown in red. Type species are shown in bold.

The combined *tef-1* and *rpb2* sequence data set was used for phylogenetic analysis of the *F. solani* species complex, following the identification techniques employed in earlier studies [50,55]. This phylogenetic analysis included 41 taxa and the aligned data set contained 1415 bp including gaps (*tef-1*: 1–603 and *rpb2*: 604–1415). The outgroup consisted of *F. decemcellulare* and *F. setosum* from the *F. decemcellulare* species complex (FDSC). A phylogenetic tree of the *F. solani* species complex is shown in Figure 3. Our phylogenetic tree was constructed with the aim of being similar to those in previous phylogenetic studies [50,55,56]. The phylogenetic tree successfully assigned the two fungal isolates (SDBR-CMU463 and SDBR-CMU464) assessed in this investigation within the same clade of *F. paranaense*, which consisted of the type species CML 1830. This clade established a monophyletic clade with high statistical support (99% BS and 1.0 PP). *Fusarium paranaense* formed a sister taxon to *F. falciforme* with high statistical support (97% BS and 1.0 PP). Thus, both fungal isolates (SDBR-CMU463 and SDBR-CMU464) were recognized as *F. paranaense*.

### 2.5. Morphological Descriptions

#### 2.5.1. *Fusarium compactum* (Wollenw.) Raillo, *Fungi of the Genus Fusarium*: 180 (1950) (Figure 4)

Colonies on OA, PDA, and SNA grew to >85.0, 25.0–3.25, and 32.0–36.0 mm in diameter, respectively, at 25 °C in the dark for one week. Colonies on PDA were yellowish white in the center, white at the margins, and flat with undulated edges that were pale yellow. Colonies on OA were greyish yellow in the center, white at the margins, had dense aerial mycelia, and were flat with entire edges that were greyish orange. Colonies on SNA were white and umbilicated with entire edges that were white. Pigment and odor were not present. Sporodochia were not found in any agar media. Conidiophores were formed on aerial mycelium, of a size of 12.5–100 × 2.8–4.2 µm, which appeared as branched, and bore terminal or lateral phialides. Phialides were monophialidic, subulate to sub-cylindrical, hyaline, smooth and thin-walled, and of a size of 13.1–31.6 × 2.6–4.3 µm. Chlamydospores were abundant, globose, ellipsoid, intercalarily or terminal, hyaline to pale yellow with age, smooth-walled, solitary, in chains or clusters, and of a size of 6.6–17.4 × 6.1–16.7 µm (av. ± SD: 11.1 ± 2.5 × 11.0 ± 2.4 µm). Microconidia were abundant, hyaline, oval to ellipsoidal, straight to slightly curved, aseptate, and of a size of 5.3–13.5 × 2.1–3.7 µm (av. ± SD: 9.6 ± 1.9 × 2.8 ± 0.3 µm). Macroconidia hyaline were thick-walled, strongly curved, had 1–7-septa, and were of a size of 13.3–72.5 × 3.3–6.4 µm (av. ± SD: 33.0 ± 13.1 × 4.6 ± 0.6 µm).

**Figure 4:**
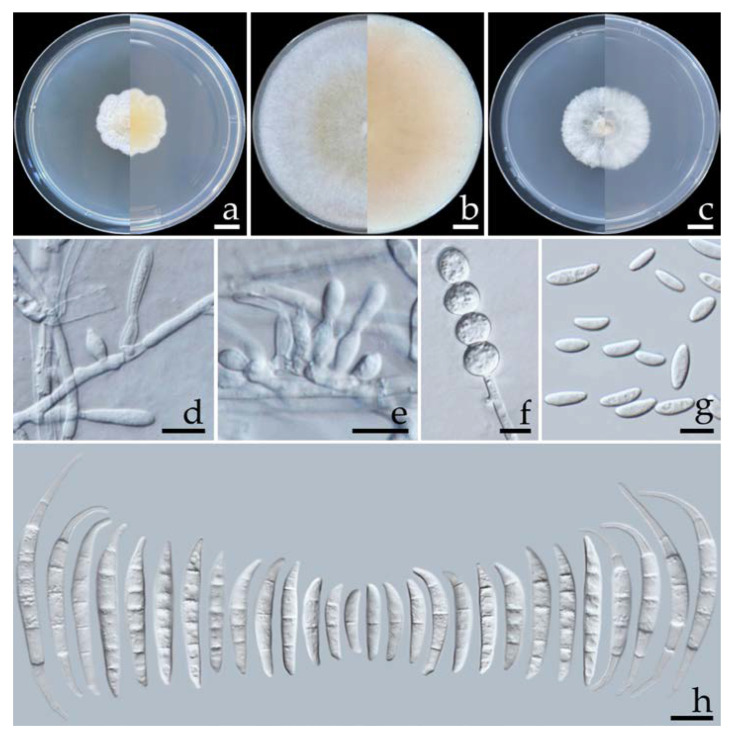
*Fusarium compactum* (SDBR-CMU461). Colony on potato dextrose agar (**a**), oatmeal agar (**b**) and synthetic nutrient-poor agar (**c**) (left, surface view and right, reverse view) after incubation at 25 °C for seven days. Phialides on aerial mycelium (**d**,**e**). Chlamydospores (**f**). Aerial microconidia (**g**). Aerial macroconidia (**h**). Scale bars: (**a**–**c**) = 10 mm; (**d**–**h**) = 10 µm.

Note: Morphologically, the two isolates of *F. compactum* obtained in this study could produce microconidia, which has not been recorded in previous studies [57,58]. However, their other morphological characteristics agreed well with the previous descriptions of F. compactum [57,58]. Phylogenetically, *F. compactum* forms a species that is phylogenetically related to F. lacertarum. However, *F. lacertarum* may be distinguished from *F. compactum* by its shorter conidiophores (up to 7.0 µm long) and phialides (2.5–4.0 × 1.0–1.5 µm) [59].

#### 2.5.2. *Fusarium paranaense* Costa, Matos & Pfenning, *Fungal Biology* 120: 55 (2015) (Figure 5)

Colonies on OA, PDA, and SNA grew to 80–83, 75.0–78.0, and 77.0–80.5 mm in diameter, respectively, at 25 °C in the dark for one week. Colonies on PDA were orange–white in the center, white at the margins, and flat with entire edges that were light yellow. Colonies on OA were brownish orange in the center and white at the margins with aerial mycelia that were dense and flat with entire edges that were brownish orange. Colonies on SNA were white and raised with entire edges that were white. Pigment and odor were not present. Sporodochia were not found in any agar media. Conidiophores were formed on aerial mycelium, of a size of 12–105 × 2.5–4.1 µm, were verticillately branched, and bore terminal or lateral phialides. Phialides were monophialidic, subulate to sub-cylindrical, hyaline, smooth and thin-walled, and of a size of 10.8–38.9 × 2.3–5.4 µm. Chlamydospores were abundant, hyaline, globose, intercalarily or terminal, ellipsoid, smooth to rough-walled, solitary, or were present in pairs or formed chains, and of a size of 6.2–11.3 × 6.2–11.6 µm (av. ± SD: 9.2 ± 1.4 × 8.9 ± 1.3 µm). Microconidia were abundant, hyaline, thin-walled, elongated to ellipsoidal, straight to slightly curved, aseptate, and of a size of 5.3–20.1 × 2.3–5.2 µm (av. ± SD: 11.4 ± 3.4 × 4.0 ± 0.7 µm). Macroconidia were hyaline, cylindrical to fusiform, 1–4-septate, and of a size of 16.0–40.6 × 3.5–5.4 µm (av. ± SD: 29.1 ± 6.5 × 4.7 ± 0.4 µm).

**Figure 5:**
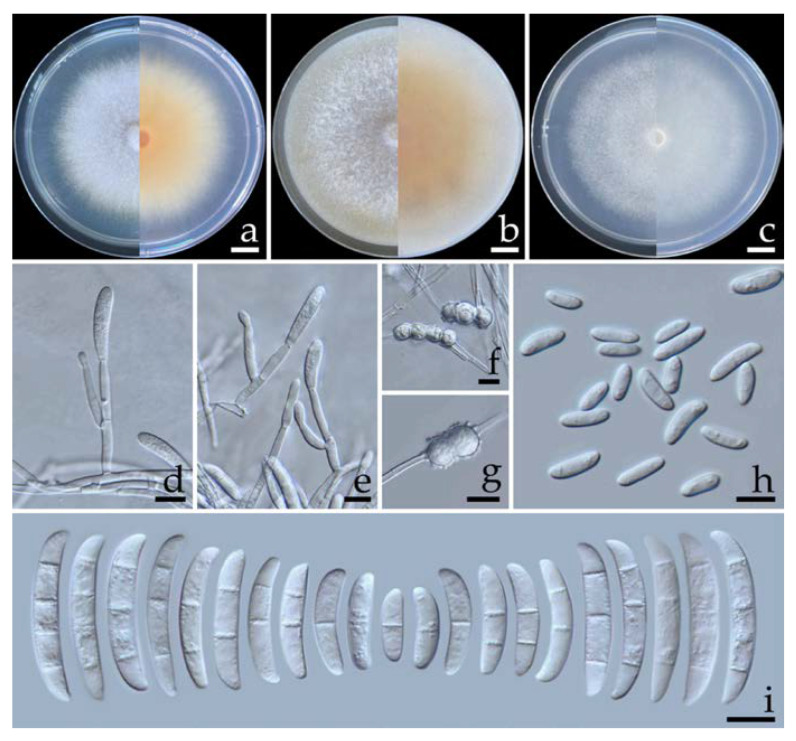
*Fusarium paranaense* (SDBR-CMU463). Colony on potato dextrose agar (**a**), oatmeal agar (**b**) and synthetic nutrient-poor agar (**c**) (left, surface view and right, reverse view) after incubation at 25 °C for seven days. Conidiophores on aerial mycelium (**d**). Phialides on aerial mycelium (**e**). Smooth and rough-walled chlamydospores (**f**,**g**). Aerial microconidia (**h**). Aerial macroconidia (**i**). Scale bars: (**a**–**c**) = 10 mm; (**d**–**i**) = 10 µm.

Note: The morphological characteristics of isolates SDBR-CMU463 and SDBR-CMU464 corresponded to descriptions of *F. paranaense* [50]. Phylogenetically, *F. paranaense* forms a sister taxon to *F. falciforme*; however, the growth of *F. paranaense* appeared to be slower than that of *F. falciforme* (85.0 mm) on PDA for one week at 25 °C [18]. In addition, *F. paranaense* produces elongated to ellipsoidal microconidia, whereas *F. falciforme* produces oval microconidia [18].

### 2.6. Pathogenicity Test

**Figure 6:**
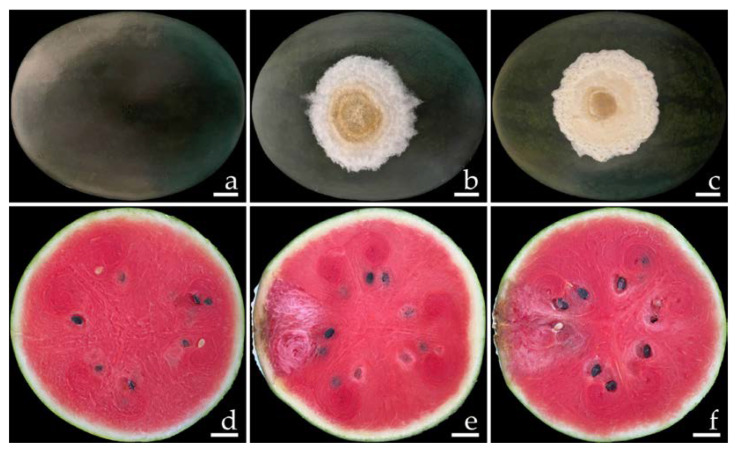
Pathogenicity test with *F. compactum* SDBR-CMU461 and SDBR-CMU462 on watermelon fruits after seven days of inoculation. Control fruit inoculated with sterile water (**a**,**d**); and disease symptoms after inoculation with isolate SDBR-CMU461 (**b**,**e**) and isolate SDBR-CMU462 (**c**,**f**). Scale bars = 20 mm.

**Figure 7:**
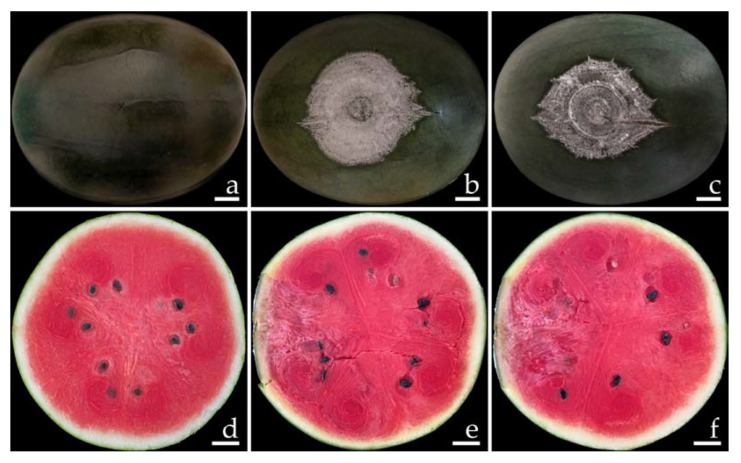
Pathogenicity test with *F. paranaense* SDBR-CMU463 and SDBR-CMU464 on watermelon fruits after seven days of inoculation. Control fruit inoculated with sterile water (**a**,**d**); and disease symptoms after inoculation with isolate SDBR-CMU463 (**b**,**e**) and isolate SDBR-CMU464 (**c**,**f**). Scale bars = 20 mm.

The disease symptoms of *F. compactum* (SDBR-CMU461 and SDBR-CMU462) and *F. paranaense* (SDBR-CMU463 and SDBR-CMU464) are shown in Figure 6 and Figure 7, respectively. Primary symptoms appeared on the wounded fruits as small light-brown to brown spots and developed into green bruises. After that, these spots developed into dark green bruised spots that were covered with a dense white mycelia for *F. compactum* (Figure 6b,c) and a thin white mycelia for *F. paranaense* (Figure 7b,c) surrounding each lesion. The inoculated fruits displayed moderate infections, as characterized by rot symptoms after one week of incubation. A cross section of a mature lesion indicated that the interior lesion area seemed to be decomposing and was encircled by water-soaked tissue (Figure 6e,f and Figure 7e,f). Following a 14-day inoculation period, the lesions covered the entire fruit. The fruits eventually became extremely rotten and squashy. The symptoms of the disease were consistent with those observed during the postharvest storage period. Nevertheless, no disease symptoms were observed on wounded fruits treated with sterile distilled water (Figure 6a,d and Figure 7a,d). Each fungal isolate was consistently re-isolated from all inoculated tissues and re-identified using both morphological methods of characterization in order to fulfill Koch’s postulates. 

## 3. Discussion

*Fusarium* is considered to be one of the most important genera of plant pathogens, as it is known to cause serious diseases in several economic plants—including watermelons—grown around the world [16,60,61]. Traditional approaches to the characterization and identification of the *Fusarium* species are mainly based on morphological characteristics [38,58,62]. Due to the wide variety of morphological differences, it is impossible to distinguish between the closely related *Fusarium* species based on morphological characteristics alone [38,58]. Therefore, molecular methods are essential to concretely identify *Fusarium* at the species level. An effective method for identifying *Fusarium* species has been designed using protein-coding (*β-tubulin*, *cam*, *tef-1*, and RNA polymerase largest sub-unit) and ribosomal DNA (the internal transcribed spacer and the large sub-unit regions) genes [35,38,42,63,64,65,66]. However, several previous studies have reported that species-level identification of *Fusarium* cannot be achieved using only the ribosomal DNA gene [67,68]. Therefore, the accurate identification of *Fusarium* species is currently carried out using a combination of morphological characteristic and multi-gene molecular phylogenetic analyses [35,36,37,38,40,63,64,66]. In this study, two isolates of *F. compactum* (SDBR-CMU461 and SDBR-CMU462) and two isolates of *F. paranaense* (SDBR-CMU463 and SDBR-CMU464) were isolated from fruit rot lesions on watermelons from northern Thailand. These four fungal isolates were identified using a combination of their morphological features and phylogenetic analysis of multiple genes, according to the identification techniques used in previous studies [35,36,37,38,50,55,56]. Prior to this study, *F. compactum* and *F. paranaense* had previously been identified as plant pathogens; for example, *F. compactum* was found to be the cause of leaf spot on sweet cherry (*Prunus avium* L.) [69] and leaf blight on maize (*Zea mays* L.) [70] in China, root rot of banana (*Musa* sp.) in Greece [71], and canker of Italian cypress (*Cupressus sempervirens*) trees in Israel [72]. In Brazil, *F. paranaense* caused root rot in soybeans [*Glycine max* (L.) Merr.] [50]. 

The pathogenicity of all *F. compactum* and *F. paranaense* isolates in this study was examined in order to confirm Koch’s postulates. According to the results, both fungal species can be regarded as causal agents of fruit rot disease in watermelons. Our results are supported by previous studies that reported that *Fusarium* species are the cause of various disease symptoms in watermelons in tropical and subtropical regions around the world [73,74,75,76]. Prior to this study, *F. solani*, *F. oxysporum*, *F. verticillioides*, and *F. chlamydosporum* were considered to be the causal agents of fruit rot in watermelons in Nigeria [16,24,77]. In particular, *F. equiseti* was found to cause fruit rot in watermelons cultivated in China [78], Malaysia [26], and the United States [79]. Postharvest fruit rot found on watermelons that was caused by *F. falciforme* and *F. oxysporum* has also been reported in Malaysia [18]. Furthermore, other *Fusarium* species have also been associated with the severity of several watermelon diseases. For example, *F. equiseti* and *F. oxysporum* f. sp. *niveum* have been observed to cause Fusarium wilt disease in fruits grown in Korea [76] and Malaysia [74], respectively. On the other hand, *Fusarium brachygibbosum* and *F. oxysporum* have been shown to lead to vine decline symptoms in the United States [73] and root rot in China [75], respectively. Furthermore, other fungal species from the genera *Alternaria*, *Aspergillus*, *Curvularia*, *Fusarium*, *Macrophomina*, *Phytophthora*, *Lasiodiplodia*, *Sclerotium*, and *Pythium* have also been associated with fruit rot in watermelons. For example, *Pythium aphanidermatum* and *P. debaryanum* caused fruit rot disease in watermelons collected in China [20]; *Phytophthora capsici* was found to cause fruit rot in watermelons in China [20] and the United States [22]; Kwon and Park [21] found that *Sclerotium rolfsii* caused postharvest fruit rot in watermelons in South Korea and, in Nigeria, *Alternaria cucumeria*, *Aspergillus flavus*, *Curvularia lunata*, *Lasiodiolodia theobromae*, and *Macrophomina phaseolina* have been identified as causal agents of postharvest fruit rot in watermelon [23,24].

In Thailand, the *Fusarium* species has been associated with symptoms of fruit rot in a number of fruits. For example, fruit rot in cantaloupes and muskmelons has been associated with *F. equiseti* [34], *F. incarnatum* [80], and *F. melonis* [13]. *Fusarium fabicercianum* caused fruit rot disease in mangoes (*Mangifera indica* Linn.) [81]. Cases of fruit rot in lychee (*Litchi chinensis* Sonn) [82] and durian (*Durio zibethinus* Murray) fruits [83] have been found to be caused by *F. solani*. Prior to this study, only incidences of watermelon fruit rot caused by *F. citrullicola* have been reported in Thailand [13]. The symptoms of fruit rot disease caused by *F. compactum* and *F. paranaense* in watermelons are similar to those determined to have been caused by known fungal pathogens [13,21,22,25]; however, to date, there have been no reports of watermelon fruit rot caused by *F. compactum* and *F. paranaense*. Therefore, we propose that *F. compactum* and *F. paranaense* should be identified as new pathogens of watermelon fruit rot in Thailand and throughout the world. Follow-up study is required to clarify the source of the disease inoculum and how weather conditions influence infection and disease development with respect to these pathogens. Furthermore, determination of the incidence of this disease in other areas of Thailand and throughout the world is a necessary task.

## 4. Materials and Methods

### 4.1. Sample Collection

Ten watermelon fruits (*Citrullus lanatus*) with typical rot symptoms were collected during the postharvest storage periods in Phayao Province, northern Thailand (19°08′20″ N, 99°54′42″ E) in 2022 (two periods: February to May and mid-October to December). All symptomatic fruits were randomly selected and placed in sterile plastic boxes. After being transported to the laboratory, the symptomatic fruits were described and assessed under a stereomicroscope (Nikon H55OS, Tokyo, Japan).

### 4.2. Fungal Isolation

All symptomatic fruits were processed to isolate the fungal causal agents by storing them in a plastic container with moistened filter paper to stimulate fungal conidia production. The single conidial isolation technique was used to isolate the causal fungi from rot lesions on 1.0% water agar supplemented with streptomycin (0.5 mg/L) under a stereomicroscope, following the methods established by Choi et al. [84]. After 24–48 h of incubation at 25 °C in the dark, individual germ conidia were selected and transferred directly onto PDA (CONDA, Madrid, Spain) including streptomycin (0.5 mg/L). Pure fungal isolates were kept in 20% glycerol and submitted to the culture collection of the SDBR-CMU, Chiang Mai Province, Thailand.

### 4.3. Pathogenicity Tests

Conidia collected from two-week-old cultures on PDA of each fungal isolate were used in this experiment. Asymptomatic commercial watermelons were thoroughly washed and their surfaces were disinfected by immersion in sterile 1.5% (*v*/*v*) NaOCl solution for 5 min. Subsequently, sterile distilled water was used to rinse them three times. After being surface-disinfected, the fruits were air-dried for 10 min at room temperature (25 ± 2 °C) [85]. The equator of each fruit received a uniform wound (5 pores, 1 mm width and 1 cm depth) with an aseptic needle after being air-dried [13]. A conidial suspension (500 µL, 1 × 10^6^ conidia/mL) of each fungal isolate was separately dropped onto the wounded fruits. Subsequently, the wounded fruits were inoculated with a drop of sterile distilled water as a control. The inoculated fruit was then kept under conditions of 80% relative humidity in a separate sterile plastic container (26 × 35.5 × 20 cm). The plastic containers were kept in a growth chamber at 25 °C during a 12 h light phase for a week. All treatments were repeated twice with ten replicates of each treatment. The samples were assessed according to the degree of disease infection on the damaged fruit areas, with scores ranging from 1–25% (mild), 26–50% (moderate), 51–75% (severe), to 76–100% (extremely severe) [86]. To confirm Koch’s postulates, the fungi were again isolated from any lesions that appeared on the inoculated fruits using the single spore isolation technique described above. The single spore isolation technique previously mentioned was employed to re-isolate the fungi from any lesions that appeared on the inoculated fruits in order to confirm Koch’s postulates.

### 4.4. Fungal Identification

#### 4.4.1. Morphological Studies

Colony characteristics of the fungal isolates on OA (Difco, Le Pont de Claix, France), PDA, and SNA were observed following incubation in darkness at 25 °C for a week, according to the methods described in previous studies [35,36,38]. Micromorphological features were assessed and photographed using a light microscope (Nikon Eclipse Ni-U, Tokyo, Japan). The size information related to the anatomical properties (e.g., chlamydospores, conidiogenous cells, conidiophores, phialides and conidia) were measured with at least 50 numbers of each structure using the Tarosoft (R) Image Frame Work program. 

#### 4.4.2. DNA Extraction, Amplification, and Sequencing

The genomic DNA of each week-old fungal isolate cultivated on PDA at 25 °C was extracted using a DNA extraction kit (FAVORGEN, Ping-Tung, Taiwan). Polymerase chain reaction (PCR) was employed to amplify the *tef-1*, *cam*, and *rpb2* genes using the primer pairs EF1/EF2 [87], CAL-228F/CAL-2Rd [88], and RPB2-5F2/RPB2-7cR [65], respectively. The three genes’ amplification programs were carried out in independent PCR reactions, consisting of an initial denaturation for 3 min at 95 °C, followed by 35 cycles of denaturation for 30 s at 95 °C, annealing steps for 50 s at 60 °C (*tef-1*), 30 s at 59 °C (*cam*) or 1 min at 52 °C (*rpb2*), and a final extension step for 1 min at 72 °C on a peqSTAR thermal cycler (PEQLAB Ltd., Fareham, U.K.). PCR products were checked and purified using a PCR clean-up Gel Extraction NucleoSpin^®^ Gel and a PCR Clean-up Kit (Macherey-Nagel, Düren, Germany), according to the manufacturer’s instructions. Following final purification, the PCR products were directly sequenced. Sequencing reactions were carried out and the above-mentioned PCR primers were employed to automatically determine the sequences in the Genetic Analyzer at the 1st Base Company (Kembangan, Malaysia).

#### 4.4.3. Sequence Alignment and Phylogenetic Analyses

The resulting *tef-1*, *cam*, and *rpb2* sequences were assessed for similarity analysis via the BLAST program available from the NCBI (http://blast.ncbi.nlm.nih.gov, accessed on 10 December 2022). Multiple sequence alignment was performed using MUSCLE [89], and any necessary modifications were made using BioEdit version 6.0.7. [90]. The combined data set of *tef-1*, *cam*, and *rpb2* data was employed to conduct a multi-gene phylogenetic analysis. Phylogenetic trees were constructed using the maximum likelihood (ML) and Bayesian inference (BI) methods. The ML analysis was performed using 25 categories and 1000 bootstrap (BS) replicates with the GTRCAT model of nucleotide substitution [91] on RAxML-HPC2 version 8.2.12 [92] at the CIPRES web portal [93]. The optimal model for substitution of nucleotides was derived using the jModeltest v.2.3 [94] according to the Akaike Information Criterion (AIC) method. BI analysis was performed using the MrBayes v. 3.2.6 software [95]. For BI analysis, six simultaneous Markov chains with random starting trees were run for a million generations, with 1000 generations of each chain being sampled. The first 2000 trees were removed using a burn-in phase, and then the remaining trees were utilized to construct a phylogenetic tree using the 50% majority rule consensus. The Bayesian posterior probabilities (PPs) were subsequently calculated. The phylogenetic trees were visualized using FigTree v1.4.0 [96].

## 5. Conclusions

Watermelon fruit rot caused by *Fusarium* species is typically spread either in the field or during storage and is occurring in many countries around the world. In the present study, we reported *F. compactum* and *F. paranaense* to be pathogens of watermelon fruit rot for the first time, in Thailand and worldwide. These fungi were obtained from rot lesions taken from watermelons and identified on the basis of morphological features and multi-gene phylogenetic analyses. In pathogenicity tests under artificial inoculation conditions, the same symptoms as those seen during the postharvest storage period were observed. Therefore, *F. compactum* and *F. paranaense* were concluded to be novel pathogens of fruit rot diseases in watermelons. Further investigation of the epidemiology of these diseases in other areas of Thailand, as well as for the purposes of establishing effective management practices, is required. Moreover, in the future, the development of efficient monitoring and preventative strategies will be necessary in order to prevent the significant financial losses introduced by fruit rot disease.

## Figures and Tables

**Table 1:** Details of the *Fusarium incarnatum-equiseti* species complex sequences used in the molecular phylogenetic analysis.

Fungal Taxa	Strain/Isolate	GenBank Accession Number			Reference
		*tef-1*	*cam*	*rpb2*	
*Fusarium aberrans*	CBS 131385 ^T^	MN170445	MN170311	MN170378	[37]
*Fusarium arcuatisporum*	LC12147 ^T^	MK289584	MK289697	MK289739	[35]
*Fusarium arcuatisporum*	LC11639	MK289586	MK289658	MK289736	[35]
*Fusarium brevicaudatum*	NRRL 43638 ^T^	GQ505665	GQ505576	GQ505843	[39]
*Fusarium bubalinum*	CBS 161.25 ^T^	MN170448	MN170314	MN170381	[37]
*Fusarium caatingaense*	URM 6779 ^T^	LS398466	−	LS398495	[40]
*Fusarium cateniforme*	CBS 150.25 ^T^	MN170451	MN170317	MN170384	[37]
*Fusarium citrullicola*	SDBR-CMU422 ^T^	OP020920	OP020924	OP020928	[13]
*Fusarium clavum*	CBS 126202 ^T^	MN170456	MN170322	MN170389	[37]
*Fusarium coffeatum*	CBS 635.76 ^T^	MN120755	MN120696	MN120736	[41]
*Fusarium coffeatum*	CBS 430.81	MN120756	MN120697	MN120737	[41]
*Fusarium compactum*	CBS 186.31 ^ET^	GQ505648	GQ505560	GQ505826	[39]
*Fusarium compactum*	CBS 185.31	GQ505646	GQ505558	GQ505824	[39]
*Fusarium compactum*	SDBR-CMU461	OQ108468	OQ108472	OQ108474	This study
*Fusarium compactum*	SDBR-CMU462	OQ108469	OQ108473	OQ108475	This study
*Fusarium duofalcatisporum*	CBS 384.94 ^T^	GQ505652	GQ505564	GQ505830	[39]
*Fusarium duofalcatisporum*	CBS 264.50	GQ505651	GQ505563	GQ505829	[39]
*Fusarium equiseti*	CBS 307.94 ^NT^	GQ505599	GQ505511	GQ505777	[39]
*Fusarium flagelliforme*	CBS 162.57 ^T^	GQ505645	GQ505557	GQ505823	[39]
*Fusarium flagelliforme*	CBS 259.54	GQ505650	GQ505562	GQ505828	[39]
*Fusarium guilinense*	LC12160 ^T^	MK289594	MK289652	MK289747	[35]
*Fusarium hainanense*	LC11638 ^T^	MK289581	MK289657	MK289735	[35]
*Fusarium incarnatum*	CBS 132.73 ^NT^	MN170476	MN170342	MN170409	[37]
* Fusarium incarnatum*	NRRL 32866	GQ505615	GQ505527	GQ505793	[39]
*Fusarium ipomoeae*	LC12165 ^T^	MK289599	MK289704	MK289752	[35]
*Fusarium ipomoeae*	LC12166	MK289600	MK289706	MK289753	[35]
* Fusarium irregulare*	LC7188 ^T^	MK289629	MK289680	MK289783	[35]
* Fusarium lacertarum*	NRRL 20423 ^T^	GQ505593	GQ505505	GQ505771	[39]
* Fusarium lacertarum*	LC7942	MK289643	MK289696	MK289797	[35]
* Fusarium longicaudatum*	CBS 123.73 ^T^	MN170481	MN170347	MN170414	[37]
* Fusarium luffae*	LC12167 ^T^	MK289601	MK289698	MK289754	[35]
* Fusarium melonis*	SDBR-CMU424 ^T^	OP020922	OP020926	OP020930	[13]
* Fusarium multiceps*	CBS 130386 ^T^	GQ505666	GQ505577	GQ505844	[39]
* Fusarium nanum*	LC12168 ^T^	MK289602	MK289651	MK289755	[35]
* Fusarium neoscirpi*	CBS 610.95 ^T^	GQ505601	GQ505513	GQ505779	[39]
* Fusarium pernambucanum*	URM 7559 ^T^	LS398489	−	LS398519	[40]
* Fusarium persicinum*	CBS 479.83 ^T^	MN170495	MN170361	MN170428	[37]
* Fusarium scirpi*	CBS 447.84 ^NT^	GQ505654	GQ505566	GQ505832	[39]
* Fusarium scirpi*	CBS 448.84	GQ505592	GQ505504	GQ505770	[39]
* Fusarium serpentinum*	CBS 119880 ^T^	MN170499	MN170365	MN170432	[37]
* Fusarium sulawesiense*	InaCC F940 ^T^	LS479443	LS479422	LS479855	[42]
* Fusarium tanahbumbuense*	InaCC F965 ^T^	LS479448	LS479432	LS479863	[42]
* Fusarium toxicum*	CBS 406.86 ^T^	MN170508	MN170374	MN170441	[37]
* Fusarium camptoceras*	CBS 193.65 ^ET^	MN170450	MN170316	MN170383	[37]
* Fusarium neosemitectum*	CBS 189.60 ^T^	MN170489	MN170355	MN170422	[37]

Ex-type, epi-type, and neotype species are indicated by the superscript letters as “T”, “ET,” and “NT,” respectively. The symbol “−“ indicates the absence of sequencing information in GenBank.

**Table 2:** Details of the *Fusarium solani* species complex sequences used in the molecular phylogenetic analysis.

Fungal Taxa	Strain/Isolate	GenBank Accession Number		Reference
		*tef-1*	*rpb2*	
* Fusarium azukicola*	NRRL 54364 ^T^	JQ670137	KJ511287	[43]
* Fusarium bataticola*	NRRL 22402 ^T^	AF178344	FJ240381	[44]
* Fusarium bataticola*	NRRL 22400	AF178343	EU329509	[44]
* Fusarium bostrycoides*	CBS 144.25 ^NT^	LR583597	LR583818	[45]
* Fusarium brasiliense*	NRRL 31757 ^T^	EF408409	EU329565	[44]
* Fusarium breve*	CBS 144387 ^T^	LR583601	LR583822	[45]
* Fusarium cuneirostrum*	NRRL 31157 ^T^	EF408414	FJ240389	[44]
* Fusarium diminutum*	CBS 144390 ^T^	LR583607	LR583828	[45]
* Fusarium diminutum*	LC13825	MW620164	MW474689	[36]
* Fusarium falciforme*	CBS 475.67 ^T^	LT906669	LT960558	[45]
* Fusarium falciforme*	NRRL 32778	DQ247088	EU329616	[44,46]
* Fusarium helgardnirenbergiae*	NRRL 22387 ^T^	AF178339	EU329505	[44]
* Fusarium hypothenemi*	NRRL 52782 ^T^	JF740850	JF741176	[47]
* Fusarium keratoplasticum*	NRRL 22661 ^T^	JN235712	JN235897	[48]
* Fusarium keratoplasticum*	NRRL 46437	GU170623	GU170588	[49]
* Fusarium liriodendri*	NRRL 22389 ^T^	AF178340	EU329506	[44]
* Fusarium metavorans*	CBS 135789 ^T^	LR583627	LR583849	[45]
* Fusarium metavorans*	CBS 143219	LR583629	LR583851	[45]
* Fusarium mori*	NRRL 22230 ^T^	AF178358	EU329499	[44]
* Fusarium mori*	NRRL 22157	AF178359	EU329493	[44]
* Fusarium paraeumartii*	NRRL 13997 ^T^	DQ247549	LR583855	[45,46]
* Fusarium paraeumartii*	LC13835	MW620180	MW474705	[36]
* Fusarium paranaense*	CML 1830 ^T^	KF597797	KF680011	[50]
* Fusarium paranaense*	CML 1993	KF597800	KF680004	[50]
* Fusarium paranaense*	SDBR-CMU463	OQ108470	OQ108476	This study
* Fusarium paranaense*	SDBR-CMU464	OQ108471	OQ108477	This study
* Fusarium parceramosum*	CBS 115695 ^T^	JX435149	JX435249	[51]
* Fusarium perseae*	CBS 144142 ^T^	LT991902	LT991909	[45]
* Fusarium pseudoradicicola*	NRRL 25137 ^T^	JF740757	JF741084	[47]
* Fusarium pseudoradicicola*	NRRL 25138	JF740758	JF741085	[47]
* Fusarium quercinum*	NRRL 22652 ^T^	DQ247634	LR583869	[38,46]
* Fusarium regulare*	CBS 230.34 ^T^	LR583643	MW834029	[38,45]
* Fusarium samuelsii*	CBS 114067 ^T^	LR583644	LR583874	[45]
* Fusarium silvicola*	CBS 123846 ^T^	LR583646	LR583876	[45]
* Fusarium solani*	NRRL 66304 ^ET^	KT313611	KT313623	[52]
* Fusarium solani*	NRRL 43474	EF452945	EF469984	[53]
* Fusarium vanettenii*	NRRL 45880 ^ET^	FJ240352	EU329640	[44]
* Fusarium waltergamsii*	NRRL 32323 ^T^	DQ246951	EU329576	[44,46]
* Fusarium yamamotoi*	NRRL 22277 ^ET^	AF178336	FJ240380	[44]
* Fusarium decemcellulare*	LC13606	MW580428	MW474374	[36]
* Fusarium setosum*	CBS 635.92 ^ET^	MW834294	JX171651	[38,54]

Ex-type, epi-type, and neotype species are indicated by the superscript letters as “T”, “ET”, and “NT”, respectively. The symbol “−” indicates the absence of sequencing information in GenBank.

## Data Availability

The DNA sequence data obtained from this study have been deposited in GenBank under accession numbers; *tef-1* (OQ108468, OQ108469, OQ108470, OQ108471), *cam* (OQ108472, OQ108473), and *rpb2* (OQ108474, OQ108475, OQ108476, OQ108477).
